# Meningoencephalitis Caused by Porphyromonas gingivalis: A Case Report and Literature Review

**DOI:** 10.7759/cureus.118027

**Published:** 2026-10-16

**Authors:** Mateo Blazevic, Christian P Kamm, Dávid M Gyurkó, Sonja Bertschy, Ariane Dampfhoffer

**Affiliations:** 1 Department of Neurology, Lucerne Cantonal Hospital, Lucerne, CHE; 2 Department of Neurology, Inselspital, Bern University Hospital, University of Bern, Bern, CHE; 3 Department of Neurology, Neurocenter Lucerne, St. Anna Clinic, Bellevue Medical Group, Lucerne, CHE; 4 Department of Infectiology, Lucerne Cantonal Hospital, Lucerne, CHE

**Keywords:** anaerobic meningitis, eubacterial pcr, meningoencephalitis, odontogenic infection, porphyromonas gingivalis

## Abstract

Anaerobic bacteria are a rare cause of adult bacterial meningitis. We report the case of a 48-year-old man who presented with fever, confusion, headache, and word-finding difficulty approximately two weeks after molar extraction performed in the setting of periodontal disease. Cerebrospinal fluid (CSF) analysis showed findings consistent with bacterial meningitis, while routine cultures remained negative. Additional broad-range eubacterial polymerase chain reaction (PCR) of the initial CSF sample detected *Porphyromonas gingivalis* DNA, leading to targeted antimicrobial therapy with ceftriaxone and metronidazole. Periodontal source control was achieved through extraction of multiple molars. The patient showed gradual neurological improvement but required prolonged inpatient rehabilitation due to persistent dizziness, ultimately achieving independent ambulation and complete resolution of headaches.

This case highlights the importance of considering a possible odontogenic source in patients presenting with meningitis following recent dental procedures. Molecular diagnostics may aid pathogen identification when CSF findings suggest bacterial infection despite negative cultures. In this patient, source control and antimicrobial therapy were followed by a favorable neurological outcome.

## Introduction

Odontogenic infections are common in the general population and typically remain confined to the oral cavity and adjacent tissues. Dental procedures may disrupt oral immune homeostasis, however, leading to contiguous or hematogenous spread of dental or periodontal infections to the central nervous system [1,2]. *Porphyromonas gingivalis *is a Gram-negative, obligate anaerobic rod and a key etiologic agent in chronic periodontitis. While its role in periodontal disease is well established, only a few cases of meningitis or meningoencephalitis caused by *P. gingivalis* have been reported in the literature [3-6].

Here, we describe a case of acute meningoencephalitis caused by *P. gingivalis* in a middle-aged man, occurring in the context of advanced periodontitis and recent molar extraction. We summarize previously published cases identified through a PubMed literature search and highlight the need to consider odontogenic sources in patients presenting with meningitis after dental procedures.

## Case presentation

A 48-year-old man presented to the emergency department of a secondary care hospital with fever up to 40°C, a deteriorated general condition, confusion, disorientation, and mild aphasia. No signs of meningism were observed. He was tachypneic and tachycardic, with blood pressure within the normal range. On admission, he exhibited mildly reduced alertness, with a Glasgow Coma Scale score of 14. His past medical history was notable for class III obesity (body weight 220 kg) without any other known comorbidities, and he was not taking any regular medication.

Symptom onset was temporally related to the extraction of a molar tooth two weeks earlier, when the dentist had already noted an inflamed periodontal pocket. The extraction was uncomplicated, with no immediate postoperative complications reported. Since the dental procedure, the patient had complained of “flu-like” symptoms, dizziness, and worsening of pre-existing headaches. Two days prior to hospitalization, he developed a fever up to 40°C; his dizziness increased, and he began dropping objects. He became unable to speak coherently and showed a confused state with somnolence.

On admission, initial laboratory tests revealed mildly elevated inflammatory markers (leukocytes, 15.1 × 10^9^/L; C-reactive protein, 29 mg/L) and a hypoosmolar hyponatremia (Table 1). He was admitted to the intensive care unit with a working diagnosis of infection of unknown origin and symptomatic hyponatremia.

**Table 1:** Initial laboratory findings on admission

Parameter	Patient result	Reference
Leukocytes (×10^9^/L)	15.1	3.7-9.2
Erythrocytes (×10^12^/L)	4.52	4.4-5.7
Hemoglobin (g/L)	131	134-170
Hematocrit (L/L)	0.37	0.41-0.50
C-reactive protein (mg/L)	29	<5
Sodium (mmol/L)	122	136-145
Potassium (mmol/L)	3.2	3.4-4.5
Serum osmolality (mOsm/kg)	256	280-300
Procalcitonin (µg/L)	0.09	<0.5
Prothrombin time (quick, %)	65	70-130
International normalized ratio (INR)	1.3	<1.3

Cerebrospinal fluid (CSF) analysis was highly suggestive of bacterial meningitis, showing a markedly elevated leukocyte count (950/µL) with predominance of polymorphonuclear cells, elevated lactate (7 mmol/L), and low glucose levels (0.6 mmol/L) (Table 2).

**Table 2:** Cerebrospinal fluid findings on day 5 of hospitalization

Parameter	Patient result	Reference
Appearance	Turbid	Clear
Glucose (mmol/L)	0.6	2.2-3.9
Lactate (mmol/L)	7.0	1.01-2.09
Protein (g/L)	4.07	0.15-0.45
Cell count (/µL)	950	0-3
Cell differentiation	67% polymorphonuclear; 33% mononuclear	-
Eubacterial polymerase chain reaction (PCR)	Porphyromonas gingivalis	-

Empirical antimicrobial therapy with ceftriaxone, amoxicillin, aciclovir, and an antituberculosis regimen was initiated, and the patient was transferred to our tertiary care center.

Due to the patient's body weight of 220 kg, which exceeded the weight limit of the available CT scanner, a native cranial CT could not be performed. Instead, only a venous-phase CT was obtained to minimize scan time, which limited its diagnostic value. This examination showed no evidence of a large intracranial mass lesion or clear meningeal contrast enhancement, although interpretation was constrained by the reduced image quality and protocol limitations (Figures 1A-1B). Magnetic resonance imaging (MRI) was recommended to exclude a cerebral abscess; however, the patient repeatedly and firmly refused MRI imaging. A repeat cranial CT scan was proposed as an alternative, but the patient declined this as well.

**Figure 1:**
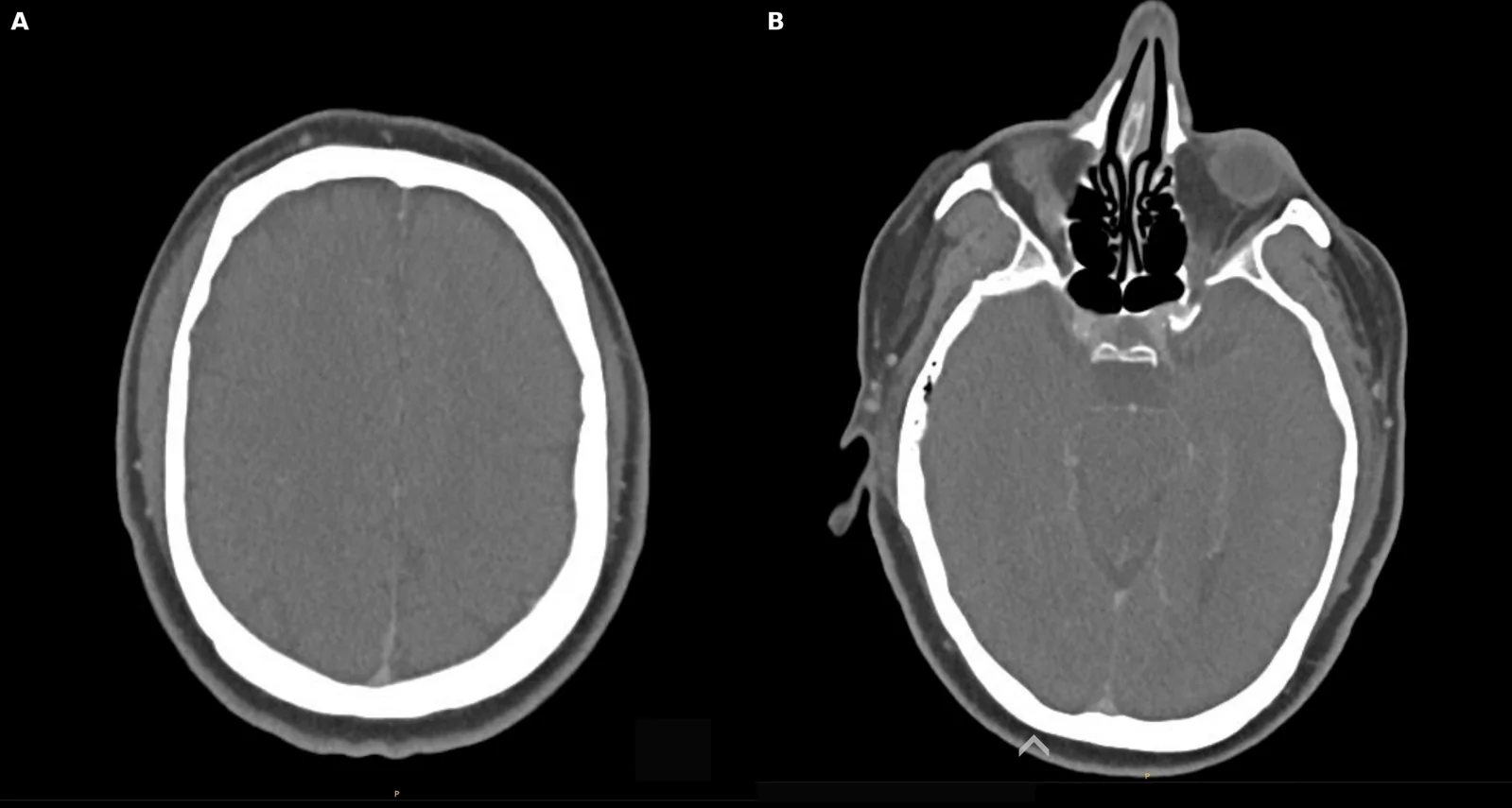
Axial venous-phase CT images (A and B) showing no evidence of a large intracranial mass lesion or clear meningeal contrast enhancement

After a negative interferon-gamma release assay (QuantiFERON) and negative *Mycobacterium tuberculosis* PCR in the CSF, antituberculosis treatment was discontinued. Mycobacterial cultures remained negative throughout the full incubation period. After exclusion of *Listeria* and herpes simplex virus infections, amoxicillin and aciclovir were also stopped, and ceftriaxone was continued. Routine cultures remained negative. However, additional broad-range eubacterial PCR of the initial CSF sample detected bacterial DNA. Subsequent sequencing and comparison with reference sequences identified *P. gingivalis* with 99.5% sequence identity, and metronidazole was therefore added to improve anaerobic coverage.

Oral and maxillofacial surgery was consulted and confirmed generalized advanced periodontitis with involvement of all molars and advanced alveolar bone loss, particularly in regions 16/17/18 and 46/47/48. Definitive dental source control was performed with extraction of teeth 18, 17, 16, 36, 46, 47, and 48, without complications. To ensure adequate perioperative coverage, antibiotic therapy with ceftriaxone and metronidazole was continued for a total of 22 and 18 days, respectively. Given the temporal association with the molar extraction, the maxillofacial findings, and the detection of *P. gingivalis* by PCR in the initial CSF sample, we diagnosed an anaerobic, odontogenic meningoencephalitis caused by *P. gingivalis*.

During the subsequent in-hospital course, the patient was awake and oriented but continued to report pronounced headache and dizziness, particularly when mobilizing from bed. Despite intensive physiotherapy, mobilization remained difficult. Overall, however, he showed a clear and continuous clinical improvement, and he was transferred to an inpatient rehabilitation facility after 34 days of hospitalization.

During rehabilitation, intensive physiotherapy and occupational therapy led to a marked improvement in mobility and gait stability. By the time of discharge, he was able to walk independently and safely on level ground both indoors and outdoors. Headaches had completely resolved. He was discharged home after 27 days of neurorehabilitation.

## Discussion

Anaerobic pathogens are a rare cause of adult bacterial meningitis (e.g., 2.4% of 540 cases) [7]. Infections caused by *P. gingivalis* are particularly uncommon, and clinical characterization is limited. We identified only four cases of meningoencephalitis associated with *P. gingivalis* (Table 3). The reported patients varied considerably in age and sex, and no common demographic characteristics could be identified.

**Table 3:** Overview of reported meningitis/meningoencephalitis-cases related to Porphyromonas gingivalis CSF: cerebrospinal fluid; PCR: polymerase chain reaction; mNGS: metagenomic next-generation sequencing

Reference	Age and gender	Medical history	Diagnosis	Aetiology	Symptoms/duration	Pathogen in CSF	Therapy	Outcome
Llitjos et al. (2016) [3]	69, F	No relevant medical history	Fatal polymicrobial purulent meningitis and severe pleocytosis with four unusual oropharyngeal bacteria	Identified periodontal abscesses without any neoplastic lesion.	Coma and hemodynamic instability	CSF culture: *Peptostreptococcus micros*, *Fusobacterium necrophorum*, *Porphyromonas gingivalis.* Eubacterial PCR: *Campylobacter rectus*	Meropenem + vancomycin + fosfomycin (initial three days); then amoxicillin + metronidazole	Patient's neurological condition worsened, and she died on day 47
Li et al. (2022) [4]	16, F	No comorbidities reported	Polymicrobial anaerobic meningitis	Revealed bone destruction at the base of the skull; speculated that sphenoid sinusitis had led to the destruction of the clivus with subsequent infection of the meninges by oral anaerobic bacteria	Fever, severe headache	mNGS: *P. gingivalis*, *Prevotella enoeca*, *C. rectus*, *Fusobacterium nucleatum*, and *Actinomyces israelii*. Repeat CSF cultures were negative	Ceftriaxone + vancomycin + metronidazole	At three-month follow-up, the patient appeared well and had returned to normal activity
Zhong et al. (2023) [5]	58, F	Chronic headache (30 years) and dental caries with intermittent toothache (six years)	Suppurative meningoencephalitis and acute cerebral infarction	Caused by infection of oral anaerobes and intra-oral abscess (gum and surrounding soft tissue infection)	Fever, headache for 21 days and left limb weakness for two days	mNGS: *Eubacterium brachy*, *P. gingivalis*, *F. nucleatum, *and torque teno virus	Mannitol, ceftriaxone (three days) + vancomycin 0.5g (16 days); subsequently ceftriaxone (two days) + metronidazole (13 days)	Symptoms improved; received rehabilitation treatment
Kiramira et al. (2024) [6]	27, M	Healthy patient	Bacterial meningitis with associated vasculitis and ischemic stroke	Resulting from bacteremia after undergoing wisdom teeth extraction within one week	Initially fever and malaise, which were followed by right-sided hemiplegia	mNGS: *F. nucleatum*, *P. gingivalis*, *Tannerella forsythia*, and *Parvimonas micra*	Ceftriaxone + ampicillin + aciclovir (initial); ceftriaxone + metronidazole (adjusted post-NGS)	Decrease in CSF cell count, lactate, and protein levels; clinical outcome not specified

Our 48-year-old male patient showed a clear temporal association between recent molar extraction and symptom onset, and maxillofacial assessment confirmed generalized advanced periodontitis with extensive alveolar bone loss, supporting an odontogenic source of infection. Oral pathogens may reach the central nervous system either via contiguous spread from adjacent structures or via hematogenous dissemination [2]. In our patient, the absence of sinus, mastoid, or middle ear pathology makes contiguous spread less likely, whereas a hematogenous route following procedure-related and/or recurrent oral bacteremia appears more plausible. Notably, although the presumed routes of infection differed among the previously reported cases of *P. gingivalis* meningoencephalitis, all were associated with pathology originating from the oral cavity or dentoalveolar region. Proposed mechanisms included periodontal and intra-oral abscesses, hematogenous dissemination following dental extraction, and contiguous spread from sphenoid sinusitis caused by oral anaerobic bacteria [3-6]. This highlights the importance of systematically assessing recent dental procedures and dental status in patients with suspected meningoencephalitis.

The clinical presentation of our case with fever, altered mental status, headache, and language disturbance, and of the four prior *P. gingivalis* cases, closely resembled that of aerobic bacterial meningoencephalitis. Likewise, CSF analysis was strongly suggestive of a bacterial etiology (Table 1), with marked neutrophilic pleocytosis, elevated lactate, and low CSF glucose, despite persistently negative routine cultures. This constellation should raise suspicion for atypical pathogens, including anaerobes, especially when there is a plausible odontogenic source. In our patient, additional eubacterial PCR of the initial CSF sample identified *P. gingivalis* and supported the diagnosis of anaerobic meningoencephalitis, together with the clinical presentation and the clear temporal link to molar extraction. This underscores the value of molecular diagnostics as a complement to conventional microbiological investigations in meningitis of unclear etiology. Anaerobic bacteria are notoriously difficult to recover in conventional cultures due to pre-analytical and analytical constraints. *P. gingivalis* in particular is known to have low culture positivity rates [8]. Therefore, when routine CSF cultures remain negative despite a CSF profile strongly suggestive of a bacterial etiology, additional molecular testing should be considered. The use of these methods, however, depends on local availability and access to specialized laboratories, which may vary across healthcare settings.

The differential diagnosis initially included tuberculous meningitis because of the clinical course and CSF profile. However, identification of *P. gingivalis* in CSF together with a convincing odontogenic source provided a more coherent explanation and guided targeted therapy.

Remarkably, our patient presented with a monomicrobial infection caused by *P. gingivalis*, whereas all four previously published *P. gingivalis* meningitis cases were polymicrobial, consistent with the broader observation that anaerobic meningitis is typically caused by multiple anaerobic pathogens [5]. This may partly reflect methodological differences, as metagenomic next-generation sequencing (mNGS) was used in three of the published literature cases instead of eubacterial PCR [4-6].

Management of anaerobic, odontogenic meningitis is not specifically addressed in current bacterial meningitis guidelines, and standard empiric adult regimens do not reliably cover oral anaerobes. In our case, ceftriaxone was continued, and metronidazole was added after the identification of *P. gingivalis* in the CSF, thereby providing targeted anaerobic coverage. There is no consensus regarding optimal treatment duration for odontogenic anaerobic meningitis. Therefore, a 14-day course of targeted antimicrobial therapy was initially chosen based on the limited available literature and extrapolation from established guidelines for acute bacterial meningitis. However, treatment was subsequently extended to a total of 22 days of ceftriaxone and 18 days of metronidazole to provide continuous antimicrobial coverage through the perioperative period until definitive dental source control was achieved. Importantly, eradication of the oral focus - here via extraction of severely affected molars in the setting of generalized advanced periodontitis - was considered a key component of management, consistent with prior reports of intracranial infections of dental origin [2].

Clinical outcomes varied considerably among the previously reported cases. One patient died despite treatment [3], one achieved complete recovery and returned to normal activities [4], and one showed clinical improvement but required rehabilitation following cerebral infarction [5]. In the fourth case [6], CSF parameters improved under therapy, although the clinical outcome was not clearly reported; notably, the disease course was complicated by vasculitis and ischemic stroke. In comparison, our patient experienced a favorable outcome despite an initially severe presentation. Following antimicrobial therapy and supportive care, neurological symptoms gradually improved; however, persistent dizziness and functional impairment necessitated inpatient rehabilitation. Over the subsequent two months, headache resolved completely, and the patient regained independent ambulation.

Several diagnostic limitations should be acknowledged. Although *P. gingivalis* DNA was detected in CSF, the presumed odontogenic source and route of dissemination were inferred from the temporal association with molar extraction and the documented advanced periodontitis, rather than directly demonstrated. Furthermore, the patient declined both MRI and repeat cranial CT, limiting assessment for a possible intracranial abscess and further characterization of cerebral involvement. The encephalitic component was inferred clinically from altered mental status, reduced alertness, and aphasia.

Although based on a very limited number of published cases, the available evidence suggests that *P. gingivalis* meningoencephalitis can be associated with substantial morbidity and may be fatal, but that favorable recovery is achievable with timely diagnosis, appropriate antimicrobial therapy, and adequate source control. Given the risk of severe neurological complications, including cerebral vasculitis and ischemic stroke reported in previous cases [5,6], prompt recognition and early intervention are particularly important to optimize outcomes.

## Conclusions

This case underscores that anaerobic meningitis caused by *P. gingivalis*, although very uncommon, should be considered in patients presenting with meningitis or meningoencephalitis symptoms, especially in the presence of advanced periodontal disease. Additional molecular diagnostic testing, such as eubacterial PCR or mNGS, should be considered as a complement to conventional microbiological investigations when CSF findings suggest bacterial infection but routine cultures remain negative. The case highlights the need for clinical awareness of a possible odontogenic source and consideration of anaerobic coverage and dental source control as part of a multidisciplinary management approach.
